# Transcriptomic Changes Induced by Deletion of Transcriptional Regulator *GCR2* on Pentose Sugar Metabolism in *Saccharomyces cerevisiae*

**DOI:** 10.3389/fbioe.2021.654177

**Published:** 2021-03-25

**Authors:** Minhye Shin, Heeyoung Park, Sooah Kim, Eun Joong Oh, Deokyeol Jeong, Clarissa Florencia, Kyoung Heon Kim, Yong-Su Jin, Soo Rin Kim

**Affiliations:** ^1^Department of Agricultural Biotechnology, Research Institute of Agriculture and Life Science, Seoul National University, Seoul, South Korea; ^2^School of Food Science and Biotechnology, Kyungpook National University, Daegu, South Korea; ^3^Department of Environment Science and Biotechnology, Jeonju University, Jeonju, South Korea; ^4^Department of Food Science, Purdue University, West Lafayette, IN, United States; ^5^Department of Food Science and Human Nutrition, University of Illinois at Urbana-Champaign, Urbana, IL, United States; ^6^Department of Biotechnology, Graduate School, Korea University, Seoul, South Korea

**Keywords:** lignocellulosic biomass, yeast metabolic engineering, transcriptomics, glucose repression, *GCR2*, pentose phosphate pathway

## Abstract

Being a microbial host for lignocellulosic biofuel production, *Saccharomyces cerevisiae* needs to be engineered to express a heterologous xylose pathway; however, it has been challenging to optimize the engineered strain for efficient and rapid fermentation of xylose. Deletion of *PHO13* (Δ*pho13*) has been reported to be a crucial genetic perturbation in improving xylose fermentation. A confirmed mechanism of the Δ*pho13* effect on xylose fermentation is that the Δ*pho13* transcriptionally activates the genes in the non-oxidative pentose phosphate pathway (PPP). In the current study, we found a couple of engineered strains, of which phenotypes were not affected by Δ*pho13* (Δ*pho13*-negative), among many others we examined. Genome resequencing of the Δ*pho13*-negative strains revealed that a loss-of-function mutation in *GCR2* was responsible for the phenotype. Gcr2 is a global transcriptional factor involved in glucose metabolism. The results of RNA-seq confirmed that the deletion of *GCR2* (Δ*gcr2*) led to the upregulation of PPP genes as well as downregulation of glycolytic genes, and changes were more significant under xylose conditions than those under glucose conditions. Although there was no synergistic effect between Δ*pho13* and Δ*gcr2* in improving xylose fermentation, these results suggested that *GCR2* is a novel knockout target in improving lignocellulosic ethanol production.

## Introduction

Lignocellulosic biofuels are renewable liquid-fuel alternatives owing to abundant feedstock availability and substantial CO_2_ emission reduction (Lynd, 2017). *Saccharomyces cerevisiae* plays an essential role in the production of lignocellulosic biofuels by fermenting lignocellulosic sugars, mainly glucose and xylose, which requires engineering of the yeast via a heterologous xylose pathway (Kim et al., 2013c; Richa et al., 2019). Current efforts on the metabolic engineering of yeast remain focused on improving the xylose fermentation yield and productivity under multiple stress conditions of lignocellulosic biomass hydrolyzates (Jeong et al., 2020; Qin et al., 2020).

Previously, an efficient xylose-fermenting strain of *S. cerevisiae* (SR8) was developed through the introduction of a heterologous xylose pathway, optimization of its expression levels, and adaptive evolution, which resulted in a loss-of-function mutation in *PHO13* (Kim et al., 2013d; Jeong et al., 2020). Continued efforts have discovered that the deletion of *PHO13* (Δ*pho13*) resulted in transcriptional and metabolic changes favorable to xylose and other C5 sugar fermentation (Kim et al., 2015; Xu et al., 2016; Ye et al., 2019). Although *PHO13* was discovered as a knockout target to improve xylose fermentation a decade ago (Ni et al., 2007; Van Vleet et al., 2008), detailed molecular mechanisms remain unelucidated. To date, the most advanced finding is that Δ*pho13* results in the transcriptional activation of non-oxidative pentose phosphate pathway (PPP) genes, which therefore facilitates xylose metabolism (Xu et al., 2016).

The present study found that the Δ*pho13* effect on xylose fermentation was not observed in a couple of strains (the YSX3 and DA24 strains; the Δ*pho13*-negative phenotype), and a loss-of-function mutation in *GCR2* was responsible. Gcr2, as a transcriptional regulator of glycolysis, enhances the CT box-dependent transcriptional activation of a Rap1-Gcr1 complex required for the expression of glycolytic genes (Huie et al., 1992). Rap1 and Gcr1 provide specific DNA-binding and activation of glycolytic and ribosomal genes, respectively (Uemura and Fraenkel, 1990; Sasaki et al., 2005). Gcr2 functions as an activation domain for the Gcr1p–Gcr2p complex mediating high level of glycolytic gene expression (Uemura and Jigami, 1992; Cha et al., 2020). However, it is unknown how the Gcr2-associated regulatory systems would function when new pathways, such as those involving the heterologous xylose-assimilating genes, are introduced.

The aim of the current study is to discover genetic factors associated with the Δ*pho13*-negative phenotype that do not affect xylose fermentation regardless of the Δ*pho13* and to identify its molecular mechanisms. The results of the present study suggest that a native transcriptional regulator Gcr2 contributed to the suboptimal xylose fermentation of engineered *S. cerevisiae*.

## Materials and Methods

### Strain Construction

All *S. cerevisiae* strains used in the present study are listed in Table 1. To construct xylose-fermenting strains, the linear expression cassette of *Scheffersomyces stipitis XYL1*, *XYL2*, and *XYL3* was used as previously described (Kim et al., 2013d). Parental strains (D452-2, JAY291, CENPK. 2-1D, and L2612) and their derivatives expressing the xylose assimilation pathway were tested for the *pho13* effect. To delete *PHO13* (GenBank accession number NC_001136) in the xylose-fermenting strains, the *pho13*:KanMX4 cassette was used as previously described (Kim et al., 2013d). To isolate spores from the KSM diploid strain, tetrad dissection was performed as previously described (Kim et al., 2017). To delete *GCR2* (GenBank accession number NC_001146), the *gcr2*::KanMX4 cassette was amplified from the genomic DNA of the BY4742 Δ*gcr2* strain (clone ID: 12013) of the Yeast Knockout Collection (Thermo Fisher Scientific, United States) by polymerase chain reaction (PCR) using SOO303/298 primers. The PCR product was purified and integrated into the genome of SR7 strain using the LiAc transformation method (Gietz and Schiestl, 2007). The resulting deletion mutant was selected on an agar medium containing 10 g/L yeast extract, 20 g/L peptone, 20 g/L glucose (YPD), 15 g/L agar, and 300 mg/mL G418 sulfate (GoldBio, St. Louis, MO, United States).

**TABLE 1:** Strains and primers used in this study.

Strains or plasmids	Relevant genotype or descriptions	References
**Strains**		
DX123	D452-2 *XYL1 XYL2 XYL3*	Kim et al., 2012
DX123 Δ*pho13*	DX123 *pho13*::KanMX4	Kim et al., 2013d
SR6	DX123 *XYL1*	Kim et al., 2013d
SR6 Δ*pho13*	SR6 *pho13*::KanMX4	Kim et al., 2013d
SR7	SR6 *XYL2 XYL3*	Kim et al., 2013d
SR7 Δ*pho13*	SR7 *pho13*::KanMX4	Kim et al., 2013d
DGX23	D452-2 *GRE3 XYL2 XYL3*	Kim et al., 2013a
DGX23 Δ*pho13*	DGX23 *pho13*::KanMX4	This study
JX123	JAY291 *XYL1 XYL2 XYL3*	Ha et al., 2013
JX123 Δ*pho13*	*pho13*::KanMX4	This study
CX123	CEN.PK 2-1D *XYL1 XYL2 XYL3*	This study
CX123 Δ*pho13*	CX123 *pho13*::KanMX4	This study
LX123	L2612 *XYL1 XYL2 XYL3*	This study
LX123 Δ*pho13*	LX123 *pho13*::KanMX4	This study
YSX3	L2612 *XYL1 XYL2 XYL3*	Jin et al., 2003
YSX3 Δ*pho13*	*pho13*::KanMX4	This study
DA24	YSX3 m*XYL1*	Ha et al., 2011
DA24 Δ*pho13*	DA24 *pho13*::KanMX4	This study
SX3-2	D452-2 *MATa* m*XYL1 XYL2 XYL3*	Kim et al., 2013b
KSM	A diploid strain of YSX3 and SX3-2	Kim et al., 2013b
BY4742 Δ*gcr2*	Yeast Knockout Collection	Thermo Fisher Scientific
SR7 Δ*gcr2*	SR7 *gcr2*::KanMX4	This study
Primers		
SOO303	CAACCCTATGCTACAAGAGCAG	*GCR2* upstream
SOO298	CGACACTAAACCCAGCTAACTC	*GCR2* downstream

### Culture Conditions

A colony of yeast cells was pre-cultured in 5 mL YPD for 24 h at 30°C and 250 rpm. The culture containing 25 or 2.5 mg cells was centrifuged at 15,000 rpm for 1 min at 4°C. The cells were resuspended in 50 mL YPD (40 g/L glucose) or YPX (40 g/L xylose) in a 250 mL Erlenmeyer flask, and the culture with an initial dry cell weight of 0.5 or 0.05 g/L was incubated at 30°C and 80 rpm. All experiments were performed in triplicates.

### Fermentation Profiles

Cell growth was monitored at 600 nm using a spectrophotometer (OD600). The concentrations of substrates and metabolites were determined by high-performance liquid chromatography (Agilent Technologies 1260 Series, Santa Clara, CA, United States) equipped with a refractive index detector using the Rezex ROA-Organic Acid H+ (8%) column (Phenomenex, Inc., Torrance, CA, United States). The column was eluted with 0.005 N H2SO4 at a flow rate of 0.6 mL/min and 50°C.

### Genome Sequencing

For single nucleotide polymorphism (SNP) discovery in the Δ*pho13*-negative strain (YSX3), the genome of the YSX3 strain and its parental strain (L2612) were resequenced as previously described (Kim et al., 2013d). Briefly, genomic DNA was prepared using the YeaStar Genomic DNA Kit (Zymo Research), and the DNA quality was evaluated on a 1% agarose gel. The genomic DNA samples were then sequenced using an Illumina HiSeq 2500 system, and the sequencing results were analyzed using the CLC Genomic Workbench (version 6.5) program for SNP detection.

### RNA-seq

For transcriptomic analysis, RNA-seq was performed as previously described (Kim et al., 2015). Briefly, RNA was extracted from exponentially growing 0.5 mg cells of the control strain (SR7) and Δ*gcr2* mutant (SR7 Δ*gcr2*) on glucose or xylose using the Qiagen RNeasy Mini Kit, and the RNA quality was evaluated using a Bioanalyzer RNA chip. The samples with high-quality total RNA were sequenced using an Illumina HiSeq 2000 system. The sequencing results were then analyzed using the CLC Genomic Workbench (version 6.5) to investigate RNA-seq quality, differentially expressed (DE) genes, and gene set enrichment analysis (GSEA). GSEA is a computational method determining statistical significance and concordant differences of defined sets of genes (Subramanian et al., 2005). Fold changes were calculated based on the total number of exon reads per kilobase of exon length per million mapped reads (RPKM) between SR7 and SR7 Δ*gcr2* strains.

### Data Availability

Whole genome sequencing data of *S. cerevisiae* L2612 and YSX3 strains are available under NCBI BioProject PRJNA689417 and BioSample accessions (SAMN17207998 and SAMN17207999). RNA-seq data of *S. cerevisiae* SR7 and SR7 Δ*gcr2* strains are available under NCBI BioProject PRJNA689538 and BioSample accessions (SAMN17208951–SAMN17208954).

## Results

### Δ*pho13*-Negative Phenotype Was Found in a Few Xylose-Fermenting Engineered Strains

**FIGURE 1:**
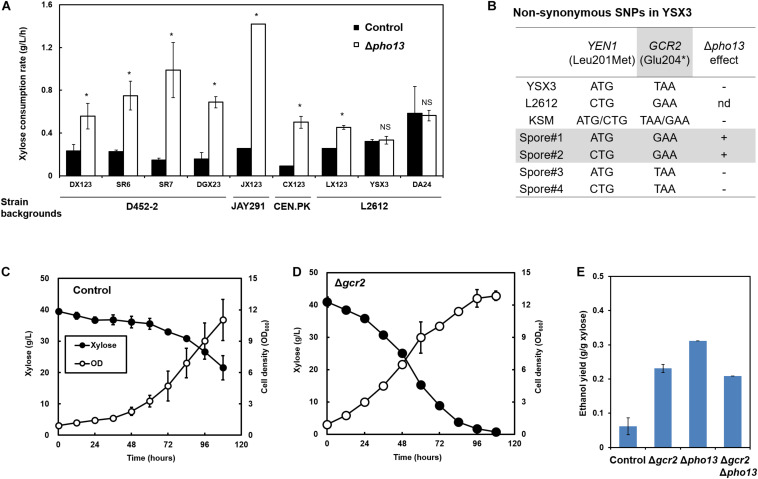
Loss-of-function mutation in *GCR2* is responsible for the Δ*pho13*-negative phenotype in xylose-fermenting *S. cerevisiae* strains. **(A)** Improved xylose consumption rates by the deletion of *PHO13* (Δ*pho13*) in engineered strains with different strain backgrounds, except for YSX3 and DA24 strains. **(B)** Genome sequencing results of the YSX3 and L2612 strains and Sanger sequencing results of haploid spores derived from the KSM diploid (YSX3 × a derivative of D452-2). **(C–E)** Xylose fermentation profiles by the SR7 strain (control) and its gene deletion mutants (Δ*gcr2*, Δ*pho13*, and Δ*gcr2*/Δ*pho13*). Fermentation was performed with an initial cell density of 0.5 g/L in YP medium containing 40 g/L xylose under microaerobic conditions. *p < 0.05; NS, not statistically significant; nd, not determined.

As previously reported, Δ*pho13* improves the xylose fermentation capability of engineered strains of *S. cerevisiae* (Ni et al., 2007; Van Vleet et al., 2008), and other studies have confirmed this with different strain backgrounds (Fujitomi et al., 2012; Kim et al., 2013d; Jeong et al., 2020). Although the detailed molecular mechanism remains unknown, Δ*pho13* causes transcriptional activation of the genes involved in non-oxidative PPP (Kim et al., 2015; Ye et al., 2019) and reduction of the dephosphorylation product of sedoheptulose-7-phosphate, suggesting the phosphatase activity of Pho13 (Xu et al., 2016). To further explore Δ*pho13*-mediated metabolic regulation, the Δ*pho13* effect was tested with a broader range of strains that we have constructed, as listed in Figure 1A and Table 1. When a plasmid expressing heterologous xylose pathway (*XYL1*, *XYL2*, and *XYL3* derived from *S. stipitis*) was introduced to four different strain backgrounds, all the resulting strains (DX123, JX123, CX123, and LX123) and their derivatives (SR6, SR7, and DGX23) showed an improved xylose consumption rate by Δ*pho13*. However, two strains, YSX3 and its derivative (DA24) did not show any improvement in xylose consumption rate by Δ*pho13*. The LX123 and YSX3 strains have been engineered to express a heterologous xylose pathway using *S. cerevisiae* L2612 as a parental strain. Thus, unlike LX123, the Δ*pho13*-negative phenotype of YSX3 can be considered independent of the strain background. Because the YSX3 strain underwent multiple transformations and vigorous screening processes for strain optimization previously (Jin et al., 2003), we hypothesized that some spontaneous mutations were acquired in YSX3, which might be responsible for its Δ*pho13*-negative phenotype.

### Loss-of-Function Mutation in *GCR2* Is Responsible for the Δ*pho13*-Negative Phenotype

To identify the molecular mechanism of the Δ*pho13*-negative phenotype of the YSX3 strain, genome sequencing of the YSX3 strain and its parental strain (L2612) was performed. Among the 44 non-synonymous SNPs identified (Supplementary Table 1), only 2 SNPs in *YEN1* (YER041W, 601C > A substitution) and *GCR2* (YNL199C, 610G > T substitution) had a high frequency (>98%) with deep coverage (172 copies), suggesting that the two SNPs are not likely sequencing errors. Sanger sequencing of the PCR-amplified genes confirmed that the two SNPs are present in YSX3 but not in L2612 (Figure 1B).

To further identify the necessity of the two SNPs in the Δ*pho13*-negative phenotype, the KSM diploid was constructed using two haploids, YSX3 (Δ*pho13*-negative) and SX3. The SX3 strain is a D452-2 derivative, in which Δ*pho13* improves its phenotypes on xylose (Δ*pho13*-positive). From tetrad dissection of the KSM strain, four spores were obtained. Based on xylose fermentation phenotypes of the spores, it was noted that two spores had the Δ*pho13*-positive phenotype, whereas the other two spores had the Δ*pho13*-negative phenotype. Sanger sequencing of the spores revealed that both Δ*pho13*-positive spores had wild-type *GCR2*, whereas Δ*pho13*-negative spores had the mutant *GCR2*. Therefore, it was concluded that the Δ*pho13*-negative phenotype is strongly associated with the mutant *GCR2* (YNL199C, 610G > T substitution).

Because the mutation in *GCR2* resulted in the truncation of the protein (Glu204^∗^), we assumed that it is a loss-of-function mutation. To test the hypothesis, the deletion of *GCR2* (Δ*gcr2*) was tested in a different strain background (SR7). Consequently, the SR7 Δ*gcr2* strain showed faster xylose consumption and higher ethanol production than the control SR7 strain (Figures 1C,D). Also, the *GCR2* deletion improved ethanol yield of xylose fermentation from 0.06 g ethanol/g xylose (SR7) to 0.23 g/g (SR7 Δ*gcr2*) (Figure 1E). However, the degree of improvement by the *GCR2* deletion was not as great as that by the *PHO13* deletion; SR7 Δ*pho13* showed the highest ethanol yield (0.31 g/g). Moreover, the *GCR2* deletion in the SR7 Δ*pho13* strain did not further improve the ethanol yield. In other words, there was no synergistic effect by double deletion of *GCR2* and *PHO13*. These results suggested that the loss-of-function mutation in *GCR2* is responsible for the Δ*pho13*-negative phenotype of the YSX3 strain. Meanwhile, it can be concluded that *GCR2* is a novel deletion target to improve xylose fermentation.

### Global Transcriptional Changes Induced by Δ*gcr2*

**FIGURE 2:**
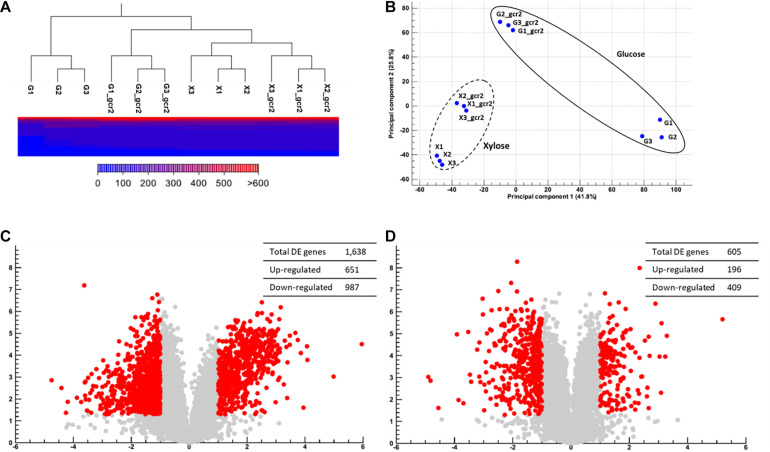
Global transcriptional changes induced by Δ*gcr2.* Hierarchical clustering and multivariate analysis based on Pearson’s correlation **(A)** and principal component analysis **(B)**. DE genes (*p* < 0.05, >2-fold) on glucose **(C)** and on xylose **(D)** were identified. RNA samples were extracted from exponentially growing SR7 and SR7 Δ*gcr2* strains on glucose and xylose.

*GCR2* encodes a transcriptional activator of glycolytic genes; therefore, its deletion leads to the transcriptional downregulation of the glycolytic genes and upregulation of the citric acid cycle genes during glucose metabolism (Uemura and Jigami, 1992; Sasaki and Uemura, 2005; Fendt et al., 2010). To investigate the transcriptional changes cause by Δ*gcr2* during xylose metabolism, *S. cerevisiae* SR7 and SR7 Δ*gcr2* strains cultured on glucose or xylose were subjected to RNA-seq, and high-quality sequencing data were obtained (Table 2). Hierarchical clustering and multivariate analysis based on Pearson’s correlation and principal component analysis, respectively, indicated that the transcriptomic profiles of glucose and xylose metabolism were the primary determinants (Figure 2). Notably, the Δ*gcr2* mutant samples were clustered separately from control samples on both glucose and xylose fermentation conditions, suggesting global transcriptional changes evoked by Δ*gcr2* regardless of the type of substrate. Meanwhile, the number of DE genes in the Δ*gcr2* mutant compared with those in the control (*p* < 0.05, >2-fold) was 1638 and 605 on glucose and xylose, respectively. Moreover, the most significant DE genes in the Δ*gcr2* mutant compared with those in the control (*p* < 0.01, >10-fold) were 17 and 5 on glucose and xylose, respectively (Table 3). The larger number of DE genes and the more significant fold changes under glucose conditions suggest that Gcr2 is responsible for higher global transcriptional regulation of glucose metabolism than that of xylose metabolism.

**TABLE 2:** Summary of RNA-seq quality, read counts, mapping rates, and transcript assemblies.

Strains and conditions	Sample name	Read count	Mapped %	Mapped to genes %
SR7 in glucose	G1	14,962,297	98.6	83.6
	G2	13,540,372	98.6	83.4
	G3	13,436,944	97.7	84.1
SR7 Δ*gcr2* in glucose	G1_gcr2	13,914,980	98.1	83.9
	G2_gcr2	13,788,675	97.9	83.4
	G3_gcr2	13,866,059	98.0	84.2
SR7 in xylose	X1	15,347,444	96.9	81.8
	X2	14,119,100	96.6	81.3
	X3	13,889,475	96.3	80.8
SR7 Δ*gcr2* in xylose	X1_gcr2	13,711,359	96.6	83.2
	X2_gcr2	13,158,020	96.7	84.4
	X3_gcr2	12,796,950	96.6	83.9

**TABLE 3:** Most significant DE genes by Δ*gcr2*^a^.

Gene name	Fold change	*p*	RPKM^b^		Molecular functions
			SR7	SR7 Δ*gcr2*	
**Glucose**					
*IMD2*	62.2	3.1E-05	19.6 ± 3.5	1217.9 ± 99.4	IMP dehydrogenase activity
*DBP2*	16.9	1.6E-04	20.9 ± 15.1	353.7 ± 39.3	ATP binding
*NOP7*	14.5	8.0E-05	17.6 ± 8.7	254.9 ± 23.4	ns
*TIP1*	12.7	9.6E-06	158.4 ± 5.4	2009.7 ± 114.1	Structural constituent of cell wall
*RPS26B*	11.1	1.3E-05	113.0 ± 30.8	1258.2 ± 69.4	Structural constituent of ribosome
*CGR1*	11.1	3.3E-05	17.1 ± 11.6	189.6 ± 8.9	ns
*NSR1*	10.5	2.1E-04	33.3 ± 20.6	350.1 ± 37.5	DNA binding
*GUA1*	10.2	4.2E-05	47.1 ± 23.6	482.5 ± 31.0	GMP synthase
*HXT5*	–10.2	3.9E-03	562.3 ± 146.1	55.1 ± 12.1	Glucose transmembrane transporter activity
*YML131W*	–10.4	7.3E-04	1401.5 ± 231.0	135.3 ± 39.1	Oxidoreductase activity
*tL(CAA)G1*	–10.7	5.4E-03	15.0 ± 4.3	1.4 ± 0.6	Triplet codon-amino acid adaptor activity
*GND2*	–11.3	1.1E-03	142.1 ± 26.5	12.6 ± 2.1	Phosphogluconate dehydrogenase
*YML089C*	–12.0	1.6E-03	15.0 ± 3.1	1.3 ± 0.3	ns
*LEE1*	–12.1	7.0E-03	265.8 ± 82.5	22.0 ± 5.0	Nucleic acid binding
*YMR206W*	–12.2	1.8E-03	117.1 ± 25.0	9.6 ± 1.1	ns
*HBN1*	–12.3	6.5E-08	207.3 ± 1.2	16.8 ± 3.2	Oxidoreductase activity
*STL1*	–27.0	1.4E-03	2746.6 ± 579.1	101.9 ± 34.3	Hydrogen symporter activity
**Xylose**					
*YDR034W-B*	–10.8	1.6E-03	181.7 ± 36.9	16.9 ± 4.6	ns
*PAU15*	–11.1	7.4E-04	16.3 ± 2.7	1.5 ± 0.6	ns
*KDX1*	–11.5	8.4E-06	347.6 ± 18.8	30.1 ± 1.6	Protein kinase activity
*DAK2*	–15.1	1.1E-05	127.0 ± 7.4	8.4 ± 1.1	Glycerone kinase activity
*ANS1*	–28.3	1.4E-03	14.5 ± 3.1	0.5 ± 0.1	ns

*^*a*^*p* < 0.01, >10-fold, range >10. ^*b*^Total number of RPKM. ns, not specific.*

### GSEA of DE Genes by Δ*gcr2*

**FIGURE 3:**
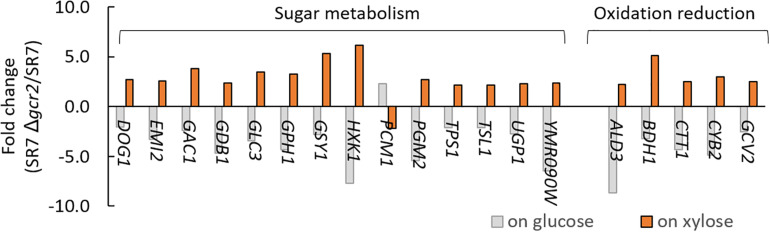
Gene sets and their genes that are oppositely affected by Δ*gcr2*. RNA samples were extracted from exponentially growing SR7 and SR7 Δ*gcr2* strains on glucose and xylose.

Differentially expressed genes in Δ*gcr2* were subjected to GSEA using Gene Ontology (GO) biological process (Table 4). Under both glucose and xylose conditions, genes associated with translation, nucleotide biosynthesis, lipid biosynthesis, and one-carbon metabolism were upregulated and those associated with protein transport were downregulated. However, the direction of the transcriptional changes by Δ*gcr2* in two gene sets (sugar metabolism and oxidation–reduction) were opposite depending on the type of substrates; they were upregulated with xylose as substrate but downregulated with glucose as substrate (Figure 3). For example, *ALD3*, encoding aldehyde dehydrogenase, is induced in response to stress; it was induced by Δ*gcr2* under xylose conditions but repressed under glucose conditions. Because Gcr2 is a native transcriptional activator for glucose metabolism, the heterologous xylose metabolism might interfere with the native metabolic regulation and cause some discrepancies in the direction of transcriptional regulation mediated by Gcr2.

**TABLE 4:** Gene set enrichment analysis (GSEA) using GO biological process on DE genes by Δ*gcr2*.

	Upregulated	Downregulated
On glucose	Translation (6) Ribosome-related (21) Nucleotide biosynthesis (1) Lipid biosynthesis (5) Antibiotic resistance (2)	Transcriptional regulation (6) Protein modification and catabolism (5) Sugar metabolism (2) Fatty acid catabolism (4) Protein transport (4) Stress response and DNA repair (7) Iron metabolism (2) Mitochondrial degradation (1) Cell division and sporulation (7) ER-associated protein catabolic process (1) Oxidation–reduction (1)
On xylose	Translation (2) Sugar metabolism (3) Nucleotide and amino acid biosynthesis (3) Lipid biosynthesis (1) Pheromone-related (2) Metabolic process (1) ER-associated protein catabolic process (1) Oxidation–reduction (1)	Protein transport (1) Cell wall organization (2)
Both	Purine nucleotide biosynthetic process (1) One-carbon metabolic process (1)	Biological_process (1)

*Numbers in parentheses represent the number of enriched gene sets.*

### Transcriptional Changes in Central Metabolic Pathways Induced by Δ*gcr2*

**FIGURE 4:**
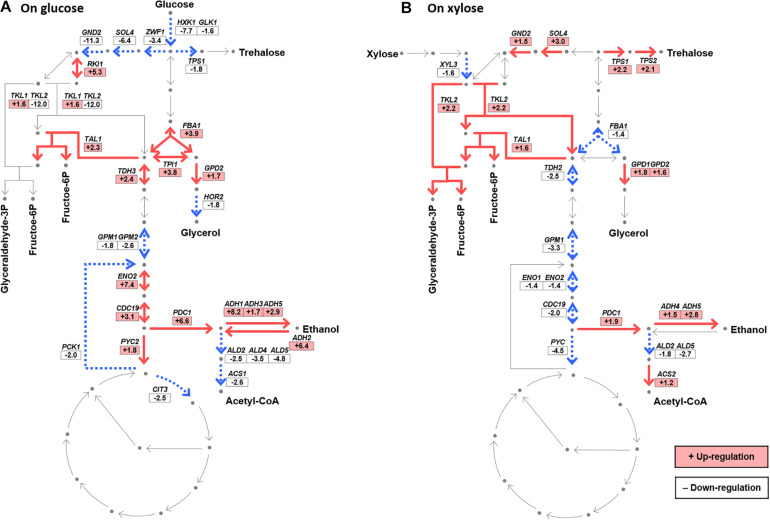
Transcriptional changes in the central metabolic pathways induced by Δ*gcr2* during glucose **(A)** or xylose **(B)** metabolism. The fold change in expression in Δ*gcr2* mutant relative to that in the wild-type strain is presented (*p* < 0.05). Glyceraldehyde-3P, glyceraldehyde-3-phosphate; fructose-6P, fructose-6-phosphate; acetyl-CoA, acetyl coenzyme A. RNA samples were extracted from exponentially growing SR7 and SR7 Δ*gcr2* strains on glucose and xylose.

To better understand the effect of Δ*gcr2* on xylose metabolism, the fold changes of DE genes (*p* < 0.05) in the glycolytic pathway, PPP, and citric acid cycle were systematically compared between glucose and xylose conditions (Figure 4). Two significant transcriptional changes were observed on both glucose and xylose conditions. First, Δ*gcr2* led to the downregulation of some glycolytic genes, most critically *GPM1* encoding phosphoglycerate mutase, which is a key enzyme of the lower glycolic pathway. Second, Δ*gcr2* upregulated non-oxidative PPP genes, most critically the *TAL1* gene encoding transaldolase. However, Δ*gcr2*-mediated transcriptional changes in central metabolic pathways were more prominent during xylose metabolism. *TDH2*, *ENO1*, and *CDC19* in the lower glycolytic pathway were significantly downregulated only under xylose conditions. Moreover, *SOL4*, *GND2*, and *TKL2* in oxidative and non-oxidative PPP were considerably upregulated only under xylose conditions. Meanwhile, down-regulation of *XYL3* was not consistent with the *XYL1*, of which expression were shared under the *TDH3* promoter.

## Discussion

In the current study, *GCR2* encoding the transcriptional activator of glycolytic genes was identified as a novel deletion target (Δ*gcr2*) to improve xylose fermentation by *S. cerevisiae*, expressing a heterologous xylose pathway. RNA-seq results revealed that Δ*gcr2* results in not only the downregulation of glycolytic genes but also the upregulation of PPP genes, which explains the improved xylose metabolism by Δ*gcr2*. However, manipulating the regulatory systems of the central metabolism such as glycolysis could result in some growth defects under certain conditions (Myung Hyun et al., 2020), which needs to be tested in follow-up studies.

Loss of function in *GCR2* leads to the Δ*pho13-*negative effect. Considering previous studies on Δ*pho13* (Kim et al., 2015; Xu et al., 2016), both Δ*pho13* and Δ*gcr2* result in the upregulation of *TAL1*, the essential overexpression target to improve xylose fermentation, as well as other genes in PPP. One hypothesis is that a simple activation signal of PPP was the main mechanism of improving xylose fermentation by Δ*gcr2* as well as Δ*pho13*; in this scenario, because PPP is already activated in the Δ*gcr2* mutant, no further effect by Δ*pho13* might be expected.

However, some interaction between Δ*gcr2* and Δ*pho13* might be possible as well. Δ*gcr2* results in more global transcriptional changes than Δ*pho13*. The number of DE genes by Δ*pho13* was 12 and 277 on glucose and xylose, respectively (Kim et al., 2015), which was one order of magnitude lower than that by Δ*gcr2*. Moreover, some transcriptional changes induced by Δ*gcr2* were in opposite directions from those induced by Δ*pho13*; especially, genes in the lower glycolytic pathway were repressed by Δ*gcr2* but activated by Δ*pho13* during xylose fermentation. Therefore, inhibition of the glycolytic pathway by Δ*gcr2* might partially counteract the Δ*pho13* effect during xylose fermentation, leading to imbalance of cofactor synthesis and/or energy generation required for xylose utilization.

Mutation of *GCR2* triggers the upregulation of oxidative and non-oxidative PPP genes. How does the up-regulated expression of oxidative PPP relate to the xylose utilization and ethanol production? A prominent property of oxidative PPP is to generate NADPH, which subsequently can be utilized by xylose reductase encoded by *XYL1* (Kwak and Jin, 2017). The increased balance between redox cofactor and the associating gene expression would promote xylose assimilation and the following non-oxidative PPP. The upregulated *TAL1* and *TKL2* genes expression suggests that native regulatory system such as Gcr2 acts negatively to the xylose fermentation, more specifically the PPP, ultimately providing the Δ*gcr2* mutant improved xylose utilization and ethanol production.

Some native regulatory systems of *S. cerevisiae* might act negatively to heterologous metabolism; however, it is challenging to systematically investigate all native regulatory genes to identify inhibitory ones. One of the most practical solutions for metabolic engineering is to use adaptive evolution to induce spontaneous mutations favorable to heterologous metabolism. Using omics approaches, such as genome sequencing and RNA-seq, our previous and present studies have identified spontaneous mutations in *PHO13* (adaptive evolution) and *GCR2* (multiple transformations and selection), which were involved in native inhibitory mechanisms against the heterologous xylose pathway.

## Data Availability Statement

The datasets presented in this study can be found in online repositories. The names of the repository/repositories and accession number(s) can be found in the article/Supplementary Material.

## Author Contributions

Y-SJ and SRK designed the experiments. MS, HP, EO, DJ, and CF carried out the experiments. SK and KK performed the statistical analysis of RNA-seq data. MS, Y-SJ, and SRK drafted and finalized the manuscript. All authors contributed to the article and read and approved the submitted version.

## Conflict of Interest

The authors declare that the research was conducted in the absence of any commercial or financial relationships that could be construed as a potential conflict of interest. The reviewer J-HS declared a shared affiliation, with no collaboration, with one of the authors MS to the handling editor at the time of review.

## Supplementary Material

Supplementary Table 1 SNPs unique to YSX3 relative to L2612.
